# High expression of cyclin D1 is associated to high proliferation rate and increased risk of mortality in women with ER-positive but not in ER-negative breast cancers

**DOI:** 10.1007/s10549-017-4294-5

**Published:** 2017-05-20

**Authors:** Cecilia Ahlin, Claudia Lundgren, Elin Embretsén-Varro, Karin Jirström, Carl Blomqvist, M. -L. Fjällskog

**Affiliations:** 1 Department of Oncology, Örebro University, Örebro, Sweden; 2 Department of Immunology Genetics and Pathology (IGP), Uppsala University, Uppsala, Sweden; 3 Department of Oncology, Helsinki University, Helsinki, Finland; 4 Department of Oncology and Pathology Lund University, Lund, Sweden

**Keywords:** Breast cancer, Proliferation, Cyclin D1, CCND1

## Abstract

**Purpose:**

Cyclin D1 has a central role in cell cycle control and is an important component of estrogen regulation of cell cycle progression. We have previously shown that high cyclin D expression is related to aggressive features of ER-positive but not ER-negative breast cancer. The aims of the present study were to validate this differential ER-related effect and furthermore explore the relationship between cyclin D overexpression and CCND1 gene amplification status in a node-negative breast cancer case–control study.

**Methods:**

Immunohistochemical nuclear expression of cyclin D1 (*n* = 364) and amplification of the gene CCND1 by fluorescent in situ hybridization (*n* = 255) was performed on tissue microarray sections from patients with T1-2N0M0 breast cancer. Patients given adjuvant chemotherapy were excluded. The primary event was defined as breast cancer death. Breast cancer-specific survival was analyzed in univariate and multivariable models using conditional logistic regression.

**Results:**

Expression of cyclin D1 above the median (61.7%) in ER breast cancer was associated with an increased risk for breast cancer death (OR 3.2 95% CI 1.5–6.8) also when adjusted for tumor size and grade (OR 3.1). No significant prognostic impact of cyclin D1 expression was found among ER-negative cases. Cyclin D1 overexpression was significantly associated to high expression of the proliferation markers cyclins A (*ρ* 0.19, *p* = 0.006) and B (*ρ* 0.18, *p* = 0.003) in ER-positive tumors, but not in ER-negative cases. There was a significant association between CCND1 amplification and cyclin D1 expression (*p* = 0.003), but CCND1 amplification was not statistically significantly prognostic (HR 1.4, 95% CI 0.4–4.4).

**Conclusion:**

We confirmed our previous observation that high cyclin D1 expression is associated to high proliferation and a threefold higher risk of death from breast cancer in ER-positive breast cancer.

## Introduction

Breast cancer is a heterogeneous disease. Decisions about adjuvant treatment have traditionally been based upon prognostic factors such as age, tumor size, histological grade, proliferation, lymph node involvement, HER2 status, estrogen receptor (ER), progesterone receptor (PgR), and gene expression assays like Oncotype DX [1]. The most commonly used proliferation marker in breast cancer hitherto has been Ki-67, although problems of method standardization cut-offs and reproducibility still remain [2].

Cyclin D1 is a member of the cyclin protein family initiated during G1 and drives the G1/S phase transition. Cyclin D1 binds to CDK4 and CDK6 and induces hyperphosphorylation of Rb, thereby promoting cellular proliferation [3]. Aberrant cyclin D1 expression is common in breast cancer [4]. Cyclin D1 expression has previously been shown to correlate strongly to ER positivity and deregulation of cyclin D1 has been associated with resistance to endocrine therapy in breast cancer cell lines [5, 6], while the role of cyclin D1 overexpression and endocrine resistance in the clinic is still controversial [5, 7–12]. The corresponding gene *CCND1* is amplified in approximately 9–30% in breast cancer [10, 13–16]. The importance of improved understanding of cyclin D1 signaling in cancer has recently been underscored due to the introduction of a new class of antineoplastic drugs, the CDK 4/6 inhibitors targeting cell cycle activation by cyclin D1 in breast cancer and other malignant diseases [3].

A large number of previous studies have investigated the prognostic impact of cyclin D1 expression or gene amplification in primary breast cancer [5, 7, 10–14, 16–49].

Most of these studies have used immunohistochemical expression of the cyclin D1 expression [7, 10, 12, 13, 16–22, 25, 26, 28, 29, 31–42, 44, 45, 47–49], some mRNA expression [5, 23, 29, 30, 46], some amplification of the CCND1 gene [10, 11, 13, 14, 16, 24, 27, 43], and several methods [10, 13, 16, 29]. There is still no consensus about which method of assessment of cyclin D1 signaling aberrations is optimal. Studies, which have used several methods of cyclin D1, have, however, shown the results of these to be significantly positively correlated. Seven studies have reported a significant positive association between CCND1 gene amplification and protein expression in addition to the present one [10, 13, 16, 28, 35, 38, 41]. Only one study [11] failed to find this association. A highly significant correlation (*ρ* 0.43, *p* < 0.0001) between cyclin D1 mRNA and protein expression has also been reported [29].

The prognostic impact of cyclin D1 amplification or overexpression in unselected breast cancer has been inconsistent; 10 studies reported cyclin D1 expression to be a favorable prognostic marker [19, 25, 28, 32, 37–39, 41, 46, 49], 8 to be unfavorable [13, 14, 18, 26, 33, 34, 42, 44], and 21 studies found no association [7, 11, 12, 16, 17, 20–24, 27, 29–31, 35, 36, 40, 43, 45, 47, 48].

Thirteen studies have studied the impact of cyclin D1 overexpression or gene amplification in ER-positive breast cancer. Six studies [5, 7, 11, 18, 42, 44] reported high cyclin D1 to be associated to higher risk of recurrence or death, while the results were non-significant in 6 [12, 13, 17, 24, 31, 45]. One study, the transATAC study, reported gene amplification to be of adverse prognostic impact, while high expression of nuclear cyclin D1 was favorable [10].

Six previous studies have analyzed the prognostic impact of cyclin D1 in ER-negative breast cancer. Overexpression was reported to be a favorable factor in two [25, 37], unfavorable in one [45], and of no prognostic impact in three studies [5, 17, 31].

In accordance with these previous inconsistent results, the recent meta-analysis by Xiao-Ling found no overall prognostic effect of cyclin D1 signaling aberrations when ER-positive and ER-negative cases were analyzed together [50]. However, cyclin D1 overexpression was consistently and significantly associated to worse prognosis in ER-positive cases.

We have previously demonstrated that high cyclin D1 expression is linked to increased proliferation in ER-positive breast cancer, while no association was seen in ER-negative disease [17]. Moreover, high cyclin D1 expression was related to shorter metastasis-free survival in patients not receiving adjuvant chemotherapy.

The aim of this study was to further explore the prognostic effect of cyclin D1 aberrations in relation to ER status in a patient material designed for evaluation of prognostic factors in early breast cancer. Furthermore, we wanted to explore the association between the protein expression of cyclin D1 and its corresponding gene, *CCND1.*

### Patients and methods

The source population of the study was a defined cohort of women diagnosed with breast cancer in the Uppsala-Örebro region during 1993–2004 as previously described [51]. The inclusion criteria were tumor size ≤50 mm, no lymph node metastases, and no adjuvant chemotherapy. From the whole cohort of 900 patients, 190 cases and 190 controls (*n* = 380) were chosen.

**Fig. 1:**
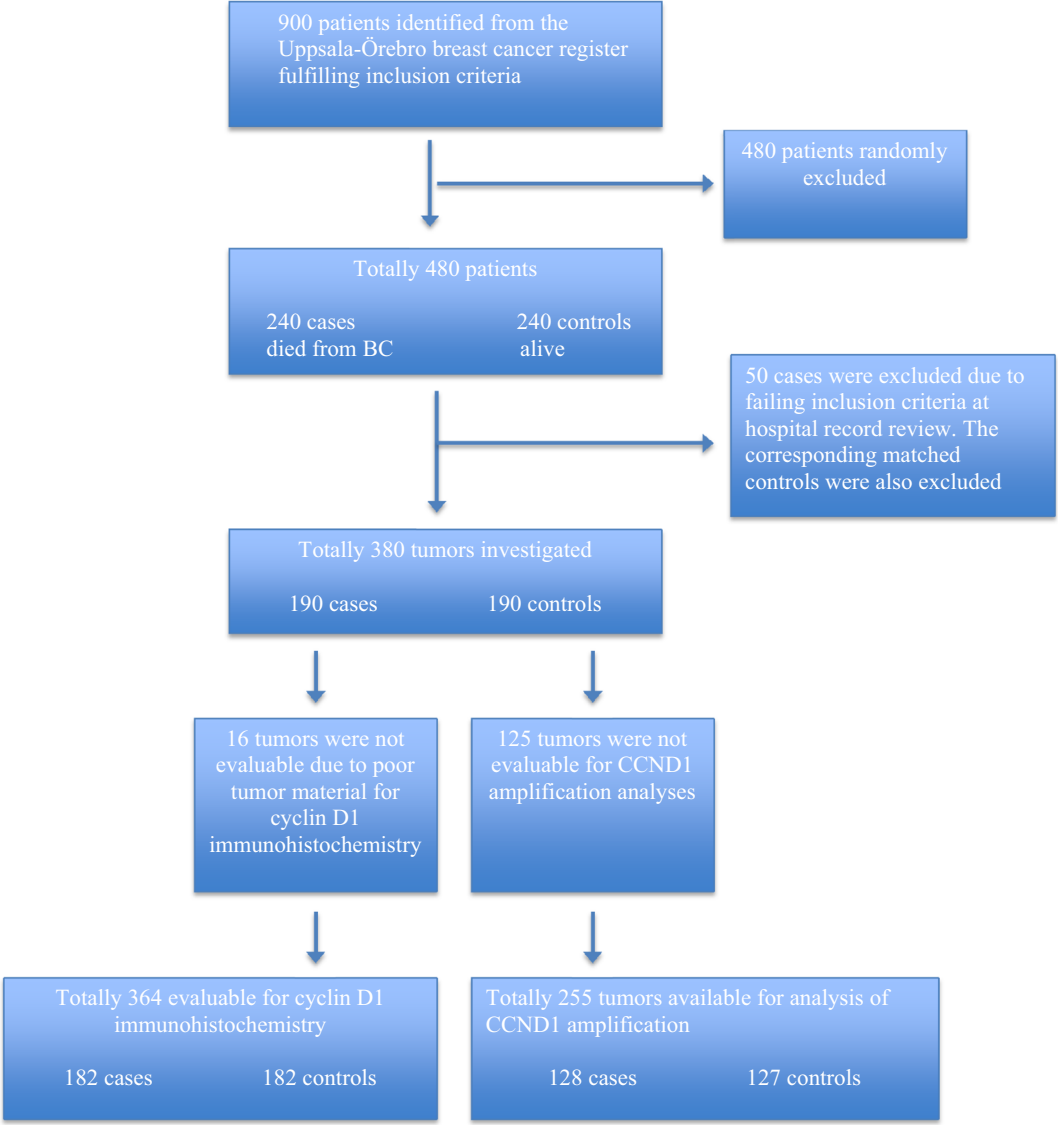
Flow chart diagram of study design

Sixteen cases were non-evaluable due to lack of tumor material, leaving 364 samples for Cyclin D1 analyses. Two hundred and fifty-five samples were evaluable for CCND1 amplification analyses (Fig. 1).

The study was approved by the local ethics committee in Uppsala, Sweden.

### TMA construction

Paraffin blocks from the patients’ primary tumors were collected. Hematoxylin and eosin sections were reviewed and areas with invasive tumor were selected. Representative areas from each tumor were punched and brought into recipient paraffin blocks to construct TMAs consisting of two cores (diameter 1 mm) of each tumor. 3–4-μm-thick sections were cut from array blocks and transferred to glass slides.

### Immunohistochemistry

TMA slides were deparaffinized in xylene and rehydrated through a ladder of graded ethanol (absolute ethanol, 95%, 80% and distilled water). For detection of cyclin D1 (RM-9104-S; NeoMarkers), antigen retrieval was performed in a microwave oven for 10 min (750 W) + 15 min (350 W) with the use of a TE (Tris–EDTA pH9 buffer). After antigen retrieval, all TMA slides were processed in an automatic immunohistochemistry staining machine according to standard procedures (Autostainer, Dako, Sweden).

### Evaluation of immunoreactivity scores

All cyclin D1 stainings were scored by one investigator (Ahlin C) blinded to all clinical information during scoring. Cells were manually counted in high-power fields. Only unequivocal nuclear staining was accepted. Hot spots were chosen for evaluation, and a minimum of 200 cells per patient were counted. Staining procedure and scoring of ER, PR, Ki-67, cyclin A, cyclin B, cyclin E, and HER2 have been described previously [51–53].

### Fluorescence in situ hybridization (FISH) analysis

For two-color FISH analysis, 4-μm-thick TMA sections were cut, mounted on positively charged glass slides (Superfrost™ Plus, Thermo Fisher Scientific) and dried. Sections were then deparaffinized in xylene, dehydrated in absolute ethanol, and subsequently pretreated using a commercial kit (Vysis paraffin pretreatment reagent kit, Abbott molecular) whereby the slides were immersed in pretreatment solution for 30 min at 80 °C followed by incubation in protease solution for 37 min at 37 °C.

FISH analyses were performed using a dual-probe kit containing an orange-labeled CCND1-specific and green-labeled CEP 11 centromere probe (Vysis CCND1/CEP 11 FISH probe kit, Abbott molecular). Hybridization and post-hybridization washes were performed according to the manufacturer’s protocol. Slides were briefly dipped in dH_2_O after being washed and ProLong^®^ Gold Antifade Mountant with DAPI (Thermo Fisher Scientific) was applied directly.

Gene-specific and centromere copy numbers were estimated by counting ≥20 nuclei in two tissue cores per case at 100x magnitude. A ratio of 1.8 or higher for CCND1/CEP was classified as CCND1 amplification. All FISH analyses were performed by one investigator (Embretsén-Varro E.) blinded to all clinical information.

### Statistical analyses

To obtain unbiased estimates of relative risk, controls were selected by incidence density sampling, which involves matching each case to a sample of those who were at risk at the time of the case occurrence.

The loss of power in comparison with a complete analysis of all cohort members is small since approximately 20% of the entire cohort was chosen as control and all eligible women with an event were included. Conditional logistic regression analysis was performed to estimate the odds ratios (ORs) and confidence interval (CI) using the proportional hazard regression procedure in statistical analysis software (IBM SPSS version 23).

Correlations of Ki 67, cyclin A, cyclin B, cyclin D, and cyclin E to other clinicopathologic parameters were evaluated using Spearman’s correlation test. Cut-off values used for cyclin A, cyclin B, cyclin E, and Ki 67 were defined as the 7th decile as previously described [51–53]. The median (61.7%) was chosen as the cut-off point for cyclin D1, but all analyses were performed also with cyclin D1 as a continuous variable.

## Results

**Fig. 2:**
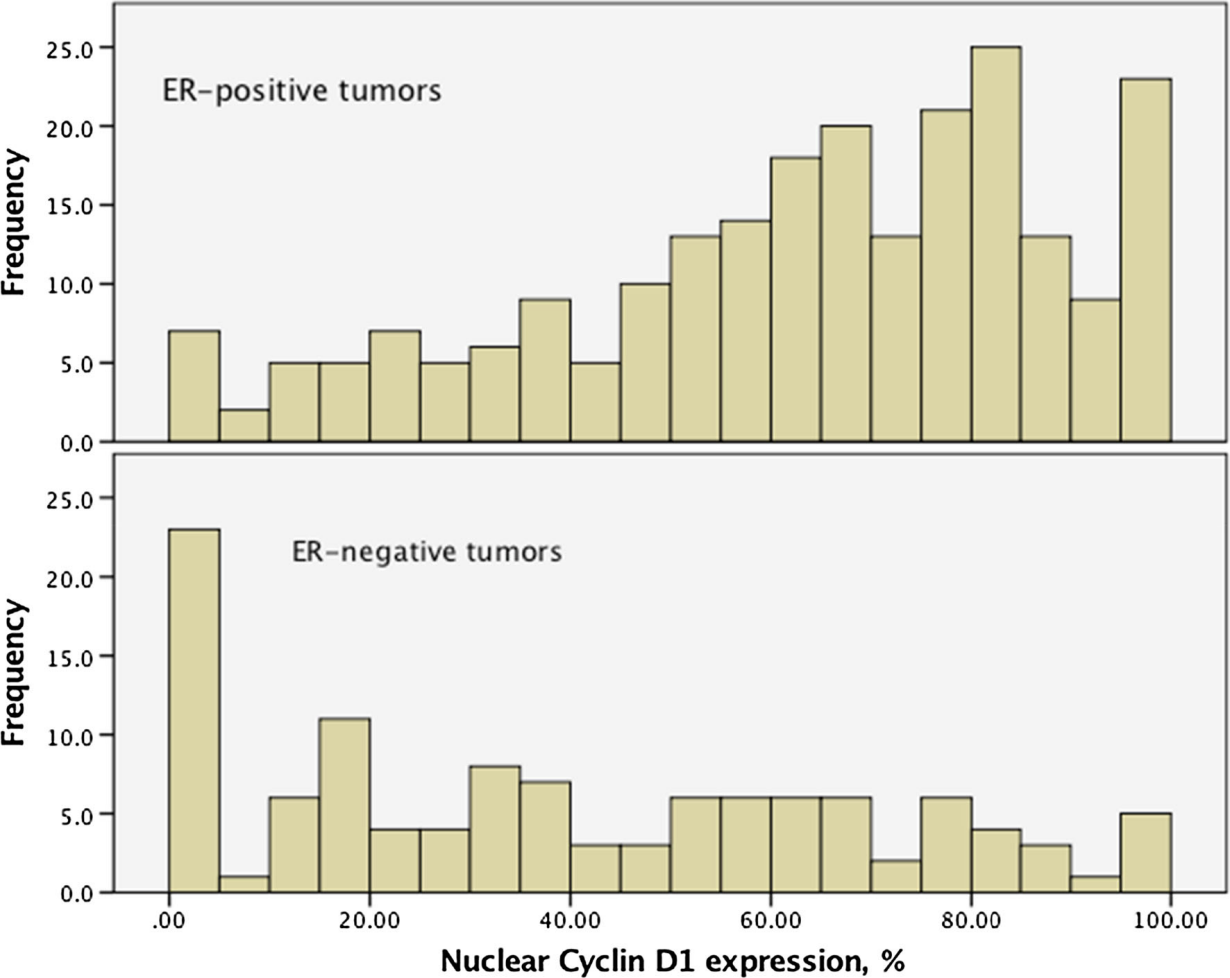
Distribution of nuclear cyclin D1 expression according to ER status

Patient and tumor characteristics according to cyclin D1 status are shown in Table 1. Median cyclin D1 expression was 61.7, 68.7% in ER-positive tumors and 34.3% in ER-negative cases. The association between ER content (percent nuclear staining) and high cyclin D1 was highly significant (Table 2). Cyclin D1 expression was below 1% in 1 of 242 ER-positive cases (0.4%) and in 6 of 115 ER-negative cases (6%). The distributions of cyclin D1 values were different in ER-positive and ER-negative tumors. Although overlapping, cyclin D1 peaked at 80% in ER-positive and less than 5% in ER-negative tumors (Fig. 2).

**Table 1:** Patient and tumor characteristics according to cyclin D1 expression

	ER status, positive >10%		ER status, negative ≤10 %		All, ER+, ER−, and ER unknown		*p* value difference between cyclin D1 high and low		
	Cyclin D low ≤ 61.7%	Cyclin D high > 61.7%	Cyclin D low ≤ 61.7%	Cyclin D high > 61.7%	Cyclin D low ≤ 61.7%	Cyclin D high > 61.7%	ER+	ER−	All
Mean age in years (range)	65 (39–89)	63 (32–87)	64 (34–88)	59 (35–80)	64 (34–89)	62 (32–87)	0.15^a^	0.09^a^	0.04^a^
Average tumor size in cm (range)	1.6 (0.4–5)	1.7 (0.2–5)	2.1 (0.6–5)	1.9 (0.3–4)	1.87 (0.4–5.0)	1.74 (0.1–5.0)	0.39^a^	0.19^a^	0.15^a^
Tumor stage, *n* (%)	93	149	85	30	182	182	0.69^b^	0.38^b^	0.04^b^
T1	71 (76)	117 (79)	46 (54)	19 (63)	121 (67)	139 (76)			
T2	22 (24)	32 (22)	39 (46)	11 (37)	61 (33)	43 (24)			
Tumor histology, *n* (%)	93	149	85	30	182	182	0.05^b^	0.06^b^	0.16^b^
Ductal	64 (69)	119 (80)	82 (97)	27 (90)	150 (82)	149 (82)			
Lobular	21 (23)	16 (11)	2 (2)	0 (0)	23(13)	16 (9)			
Others	8 (8)	14 (9)	1 (1)	3 (10)	9 (5)	17 (9)			
Histological grade, *n* (%)	92	148	84	30	180	180	0.26^c^	0.01^c^	0.01^c^
1	21 (23)	28 (19)	4 (5)	7 (23)	25 (14)	35 (19)			
2	59 (64)	93 (63)	27 (32)	9 (30)	89(49)	104 (58)			
3	12 (13)	27 (18)	53 (63)	14 (47)	66 (37)	41 (23)			
Postoperative radiotherapy, *n* (%)	93	149	85	30	182	182	0.15^b^	0.39^b^	0.46^b^
Yes	60 (65)	82 (55)	49(58)	20 (67)	111 (61)	104 (57)			
No	33 (36)	67 (45)	36 (42)	10 (33)	71(39)	78 (43)			
Endocrine therapy, *n* (%)	93	149	85	30	182	182	0.21^b^	0.52^b^	0.30^b^
Yes	34 (37)	43 (29)	22 (26)	6 (20)	124 (68)	133 (73)			
No	59 (63)	106 (71)	63 (74)	24 (80)	58 (32)	49 (27)			
PgR status, *n* (%)	93	148	85	30	178	178	0.93^b^	0.03^b^	0.001^b^
Positive >10 %	68 (73)	109 (74)	9 (11)	8 (27)	77 (43)	117 (66)			
Negative ≤10 %	25 (27)	39 (27)	76 (89)	22 (73)	101 (57)	61 (34)			
HER 2 status, *n* (%)	86	136	82	28	171	166	0.56^b^	0.54^b^	0.22^b^
Overexpression (IHC 3+ and/or FISH pos.)	3 (4)	7 (5)	16 (19)	4 (14)	19 (11)	12 (7)			
Normal	83 (97)	129 (95)	66 (81)	24 (86)	152 (89)	154 (93)			
CCND1 amplified, *n* (%)	61	99	64	25	127	124	0.02^b^	0.22^b^	0.03^b^
No	61 (100)	91(92)	61 (95)	22 (88)	124 (98)	113 (91)			
Yes	0	8 (8)	3 (5)	3 (12)	3 (2)	11 (9)			

^a^ *p* value calculated using Student’s t test

^b^ *p* value calculated using Pearson Chi-square test

^c^ *p* value calculated using Chi-square test for trend

Reference Ahlin [51]

**Table 2:** Correlation of ER and cyclin D1 to grade and proliferation markers

	ER %, all patients		D1, all patients		D1 ER-pos. group *n* = 242		D1 ER-neg. group *n* = 115	
	*ρ* ^b^	*p* value	*ρ*	*p* value	*ρ*	*p* value	*ρ*	*p* value
Ki 67^a^	**−0.17**	0.001	**−0.18**	0.001	0.08	0.241	**−**0.12	0.221
Histological grade^b^	**−0.30**	<0.001	**−0.18**	0.001	0.02	0.795	**−**0.15	0.110
Cyclin A^a^	**−0.30**	<0.001	**−0.13**	0.013	**0.18**	0.006	**−**0.08	0.400
Cyclin B^a^	**−0.23**	<0.001	**−**0.10	0.053	**0.19**	0.003	**−**0.12	0.216
Cyclin E^a^	**−0.24**	<0.001	**−0.17**	0.001	0.11	0.084	**-0.21**	0.025
Cyclin D1	**0.40**	<0.001						

^a^Pearson correlation

^b^Spearman correlation

References Ahlin [51], Nimeus-Malmstrom [52], Lundgren[53]

Significant associations (*p* < 0.05) in bold

### Correlation between ER, cyclin D1, and proliferation markers including grade

High cyclin D1 expression was significantly associated to PgR positivity and lower grade (Table 1) and ER positivity (Table 2) when ER-positive and ER-negative cancers were tested together. The associations to PgR positivity and low grade were significant only in ER-negative tumors.

A high ER receptor content was significantly associated to low tumor grade, low Ki-67 expression, and low levels of cyclins A, B, and E. In contrast, cyclin D1 showed a positive correlation to ER receptor content (Table 2).

In ER-positive tumors, high cyclin D1 expression showed a significant correlation to high expression of cyclin A and cyclin B. The correlations between cyclin D1 and other proliferation-associated factors were weakly and mostly non-significantly negative in ER-negative tumors (Table 2). The negative correlation between cyclin D1 and cyclin E in ER-negative tumors, however, reached significance (*ρ* −0.21, *p* = 0.025).

### Prognostic effect of cyclin D1 expression

Cyclin D1 expression was not significantly associated to breast cancer mortality in the study population when ER-positive and ER-negative cases were analyzed together (OR 0.94, 95% CI 0.63–1.4, *p* = 0.76).

However, in the ER-positive group, high cyclin D1 expression had a significant and strong negative effect on breast cancer mortality in both univariable (OR 3.2) and multivariable (OR 3.1) analyses (Table 3). Cyclin D1 expression remained significantly associated to breast cancer mortality in ER-positive cases also when analyzed as a continuous variable in univariable (*p* = 0.01) and multivariable (*p* = 0.03) analyses (data not shown).

**Table 3:** Uni- and multivariable analyses of breast cancer mortality

	Univariable analysis			Multivariable analysis (adjusted for tumor size, grade, and adjuvant endocrine treatment^a^)		
	OR	95% CI	*p* value	OR	95% CI	*p* value
ER-positive group *n* = 242						
Cyclin D1 (reference ≤ median, 61.7%)	**3.2**	1.5–6.8	0.002	**3.1**	1.3–7.1	0.009
Tumor size (cm)	**1.9**	1.2–3.1	0.009	**2.3**	1.2–4.2	0.01
Elston grade	**2.6**	1.3–5.0	0.005	**2.6**	1.2–5.5	0.01
Adjuvant endocrine treatment (reference: none)	0.8	0.4–1.6	0.49	0.4	0.2–1.1	0.07
ER-negative group (*n* = 115)						
Cyclin D1 (reference ≤ median, 61.7%)	0.25	0.03–2.2	0.58	0.3	0.1–2.4	0.23
Tumor size (cm)	1.6	0.6–3.9	0.34	1.6	0.6–4.3	0.31
Elston grade	1.2	0.5–2.8	0.67	1.2	0.5– 3.0	0.77

*CI* confidence interval, *OR* odds ratio

^a^For endocrine treatment in ER-positive group only

Reference Ahlin [51]

The prognostic effect of cyclin D1 expression in ER-positive cases was seen both in patients with (OR 2.0, 95% CI 0.37–10.9, *p* = 0.4, *n* = 77) and without (OR 4.0, 95% CI 1.1–14.2, *p* = 0.03, *n* = 165) adjuvant endocrine therapy.

### CCND1

The median (1.13) and mean (1.24) values of CCND1 copy number were close to 1 in most cases. No tumor had a quotient clearly less than 1. Only five tumors had a value smaller than 1, and the lowest quotient was 0.91. Fourteen tumors (5%) had a quotient higher than or equal to 1.8 and were considered to have amplification of the CCND1 gene. The highest quotient was 4.35.

### Correlation of CCND1 amplification and tumor characteristics

There was a statistically significant association between CCND1 amplification and high expression of cyclin D1 (*p* = 0.003). Mean cyclin D1 expression was 55% in tumors with normal copy number of CCND1 and 78% in the 14 amplified cases. A significant association between CCND1 amplification and cyclin D1 expression was found in both ER-positive (*p* = 0.003) and ER-negative tumors (*p* = 0.05). No significant association was found between gene amplification and tumor grade or proliferation markers (Table 4).

**Table 4:** Association between CCND1 amplification and tumor characteristics

	CCND1 amplified	CCND1 normal	*p* value
Grade			
1	1 (7%)	40 (17%)	0.14
2	6 (43%)	123 (51%)	
3	7 (50%)	77 (32%)	
Tumor size			
<2 cm	9 (64%)	160 (66%)	0.32^a^
>2 cm	5 (36%)	81 (34%)	
Histology			
Ductal	11 (79%)	208 (86%)	0.67
Lobular	1 (7%)	14 (6%)	
Other	2 (14%)	19 (8%)	
ER			
>10 %	8 (57%)	155 (65%)	0.56
≤10 %	6 (43%)	84 (35%)	
PR			
>10 %	9 (64%)	131 (55%)	0.50
≤10 %	5 (36%)	107 (45%)	
Ki-67			
<22 %	10 (71%)	146 (61%)	0.73^a^
>22 %	4 (29%)	93 (39%)	
Cyclin A			
<11 %	4 (29%)	121 (51%)	0.96^a^
>11 %	10 (71%)	117 (49%)	
Cyclin B			
<6 %	4 (29%)	102 (43%)	0.14^a^
>6 %	10 (71%)	135 (57%)	
Cyclin E			
<20.4 %	7 (50%)	138 (59%)	0.73^a^
>20.4 %	7 (50%)	98 (42%)	
Cyclin D1			
<77.8 %	5 (36%)	174 (73%)	0.003^a^
>77.8 %	9 (64%)	63 (27%)	

^a^Student’s *t* test, all other significances tested by the *χ* ^2^ test

References Ahlin 51] , Nimeus-Malmstrom[52], Lundgren [53]

### Prognostic effect of CCND1 amplification

*CCND*1 amplification had no significant prognostic impact on breast cancer mortality in all cases together (OR 1.4, 95% CI 0.4–4.4, *p* value 0.56) or in ER-positive and ER-negative cases separately (data not shown).

## Discussion

In a previous study in unselected cases of breast cancer, we found that the prognostic impact of cyclin D expression was dependent on ER receptor status [17]. In ER receptor-positive disease, high cyclin D expression was associated to high proliferation and other markers of tumor aggressiveness, while the opposite was true in ER-negative cases. Moreover, high cyclin D1 was a significant adverse prognostic factor for metastasis-free survival in chemotherapy-naïve patients with ER-positive tumors. The primary aim of the present study was to confirm these findings. Additionally, since amplification of the cyclin D gene CCND1 is one of the reasons for high cyclin D1 expression in breast cancer, we also studied the copy number of CCND1, and its association to cyclin D1 expression and prognosis.

The patients included in this study [51] were selected from the regional cancer registry of Uppsala-Örebro region in order to optimize the analysis of prognostic markers, especially cyclins and other proliferation markers in early breast cancer. Cases given adjuvant chemotherapy were excluded, since there is evidence that adjuvant chemotherapy may interfere with the prognostic effect of proliferation, possibly due to a better effect of chemotherapy in proliferating cells [17, 54].

Cyclin D1 expression was strongly associated to ER positivity in accordance with numerous previous reports [5, 12, 13, 15, 19, 27, 28, 31, 37–41, 43, 45–47, 49, 55–58]. Many cases with high cyclin D1 expression were nevertheless also found in ER-negative tumors. Low expression of cyclin D1 was almost exclusively seen in ER-negative cases.

This study confirms that the prognostic impact of cyclin D1 expression indeed depends on ER status. High expression increased breast cancer mortality in ER-positive cases, while no significant impact was seen in ER-negative cases. In ER-positive cases, cyclin D1 expression was significantly associated to markers of high proliferation including cyclins A and B. No such association was found in ER-negative tumors. On the contrary, like in our previous study [17], there was even an inverse association between the expression of the cyclins D1 and E. An early study by Kenny et al., analyzing cyclin D1 mRNA expression, came to similar conclusions, showing that cyclin D1 mRNA levels were significantly associated to relapse and breast cancer death in ER-positive disease, while no association was found in ER-negative disease [5]. This indicates that high cyclin D1 expression is linked to an activated cell cycle and worse prognosis of breast cancer in ER-positive disease, while cyclin D1 expression does not associate to markers of cell cycle activation or prognosis in ER-negative tumors. Many previous studies focusing on ER-positive breast cancer have found high cyclin D1 expression or gene amplification to be an adverse prognostic sign [5, 7, 11, 18, 42, 44, 50], while the results in ER-negative and in unselected breast cancer patients have been highly inconsistent. For further details, see the Introduction section.

One study, the TransATAC study, has reported results partly at odds with our results and other studies on ER-positive breast cancer [10]. The TransATAC study is a biomarker study on histological samples collected from patients participating in the ATAC trial, which is a randomized trial comparing anastrozole to tamoxifen adjuvant treatment in primary breast cancer. In the TransATAC study, cases with high cyclin D1 expression had a significantly longer time to recurrence and better overall survival in both univariable and multivariable analyses. However, like our study high expression of cyclin D1 was associated to gene amplification of the CCND1 and a high proliferation assessed by Ki-67 IH. Both Ki-67 expression and CCND1 gene amplification were, in contrast to cyclin D1 IH expression, associated to a worse outcome. The reason for these partly internally inconsistent associations and the difference between the results of the TransATAC study and those of our as well as the meta-analysis study are not obvious nor did the authors of the TransATAC publication suggest any explanation. TransATAC is by far the largest published study of cyclin D1 signaling and breast cancer prognosis (*n* = 1155). Thus, it is improbable that these discrepancies are due to chance alone. Several differences between the TransATAC and the current studies may offer at least a partial explanation. In contrast to our study, all patients received adjuvant endocrine treatment. Although the TransATAC analysis was restricted to ER-positive cases, the definition of ER positivity was based on the Allred score with a cut-off for positivity of 2, which corresponds to as few as 1–10% weakly positive cells [59, 60]. In our studies, we used a cut-off of 10% according to Scandinavian practice and the results of the EBCTCG meta-analysis [61]. Visual inspection of the cumulative recurrence rate curves in the TransATAC study indicated that high recurrence rate in cases with very low cyclin D1 expression (<1%) seems to account for most of the prognostic impact of cyclin D1 expression. In our study, most of the cases with cyclin D1 expression below 1% were ER negative. Thus, one may speculate that one reason for the discrepancy may be different ER receptor classification. Interestingly, the curves depicting the recurrence rate in cases with cyclin D1 expression 1–9%, 10–30%, 30–67%, and >67% in the TransATAC did not show any orderly linear association between prognosis and cyclin D1 expression level. Patients with totally negative cyclin D1 expression had the worst prognosis. However, the second worst prognosis was found in patients having high, 30–67%, cyclin D1 expression. This suggests that the association between cyclin D1 and prognosis in ER-positive breast cancer may not be simply linear. A Swedish study investigating cyclin D1 and benefit of adjuvant tamoxifen may support this interpretation; the benefit of adjuvant tamoxifen was restricted to the group with intermediate cyclin D1 expression, and these patients had the best prognosis while the patients with either very high or low cyclin D1 expression did not benefit and had a worse prognosis after endocrine treatment [12].

In conclusion, despite the partly discordant results of the large transATAC study, most previous analyses of the impact of cyclin D1 expression in ER-positive breast cancer have, like the present one, shown high expression to be a sign of tumor aggressiveness and poor prognosis.

A few previous studies have suggested that high cyclin D1 expression may be associated to tamoxifen resistance [7, 42], which might partly explain the negative impact of cyclin D1 in ER-positive breast cancer. We therefore tested the prognostic impact of cyclin D1 expression separately in patients treated or not with adjuvant tamoxifen, but found no indication that the prognostic effect was restricted to adjuvant tamoxifen use; on the contrary, the impact of cyclin D1 expression was even stronger in the group that has not received hormonal treatment. This issue is still controversial, since some studies have found cyclin D1 overexpression to be associated to tamoxifen resistance [5, 7, 8, 12], some to improved effect of endocrine treatment [7–9, 42], and some studies found no association [10, 11]. Although we cannot exclude the possibility that part of the negative impact of cyclin D1 overexpression might be explained by endocrine treatment resistance, the significant association between high cyclin D1 expression and aggressive biological features of the tumor at diagnosis indicates that adjuvant treatment resistance cannot be the only explanation.

The proportion of patients with amplification of CCND1 in the present study was low (5%), in accordance with previous large studies (*n* = 613, 738, 1155) where between 8.7 and 10% of cases have been found to have amplification [10, 13, 14]. Other smaller studies (*n* = 93, 117) have published higher frequencies of CCND1 amplification, 24.4 and 30%, respectively [15, 16]. Amplification of CCND1 thus seems to explain only a small part of cases with high cyclin D1 expression. We did not find CCND1 amplification prognostic possibly due to statistical power, since the number of amplified cases was low and confidence levels were wide (1.4, 95% CI 0.4–4.4). Seven previous studies have tested the prognostic impact of CCND1 amplification, of which two reported a significant association between amplification and worse prognosis [10, 14], one reported a significant association in ER-positive cases only [13], while five studies failed to find a significant association [11, 16, 24, 27, 43].

Our results support previous studies on the close link between ER signaling and cyclin D1 in cell cycle activation [62]. Moreover, a recent publication [63] demonstrated that cyclin D1 overexpression increased stem cell-like behavior and migration in ER-positive breast cancer cell lines, while the opposite was true in ER-negative cells [17], indicating that the effect of cyclin D1 expression in ER-positive and ER-negative breast cancer may be fundamentally different. Inhibition of the cyclin D kinases CDK4/6 by palbociclib had selective antitumor efficacy in ER-positive cell lines, while non-luminal/basal subtypes were resistant [64].

Our results may have implications for drugs targeting cyclin D1. The first phase III studies on the CDK4/inhibitors palbociclib [65, 66] or ribociclib [67] have shown impressive results in ER-positive breast cancer combined with letrozole [66, 67] or fulvestrant [65]. We have found only little data concerning the effect of CDK4/6 inhibition in ER-negative breast cancers; a phase II study of palbociclib monotherapy in advanced heavily pretreated breast cancer recruited four cases with triple-negative disease, none of which experienced a response [68].

In conclusion, this study showed that high expression of cyclin D1 is associated to cell cycle activation and poor prognosis in ER-positive tumors only. High cyclin D1 expression was significantly linked to the expression of ER and gene amplification of CCND1, although only a small proportion of cases overexpressing cyclin D1 could be attributed to gene amplification. Although a substantial proportion of ER-negative tumors also express high levels of cyclin D1, the biological role of cyclin D1 signaling in tumors lacking ER, if any, remains to be explored.
